# Oral Factors Affecting Titanium Elution and Corrosion: An In Vitro Study Using Simulated Body Fluid

**DOI:** 10.1371/journal.pone.0066052

**Published:** 2013-06-07

**Authors:** Hideki Suito, Yuki Iwawaki, Takaharu Goto, Yoritoki Tomotake, Tetsuo Ichikawa

**Affiliations:** Department of Oral and Maxillofacial Prosthodontics and Oral Implantology, Institute of Health Biosciences, The University of Tokushima, Tokushima, Japan; University of Akron, United States of America

## Abstract

**Objectives:**

Ti, which is biocompatible and resistant to corrosion, is widely used for dental implants, particularly in patients allergic to other materials. However, numerous studies have reported on Ti allergy and the *in vitro* corrosion of Ti. This study investigated the conditions that promote the elution of Ti ions from Ti implants.

**Methods:**

Specimens of commercially pure Ti, pure nickel, a magnetic alloy, and a gold alloy were tested. Each specimen was immersed in a simulated body fluid (SBF) whose pH value was controlled (2.0, 3.0, 5.0, 7.4, and 9.0) using either hydrochloric or lactic acid. The parameters investigated were the following: duration of immersion, pH of the SBF, contact with a dissimilar metal, and mechanical stimulus. The amounts of Ti ions eluted were measured using a polarized Zeeman atomic absorption spectrophotometer.

**Results:**

Eluted Ti ions were detected after 24 h (pH of 2.0 and 3.0) and after 48 h (pH of 9.0). However, even after 4 weeks, eluted Ti ions were not detected in SBF solutions with pH values of 5.0 and 7.4. Ti elution was affected by immersion time, pH, acid type, mechanical stimulus, and contact with a dissimilar metal. Elution of Ti ions in a *Candida albicans* culture medium was observed after 72 h.

**Significance:**

Elution of Ti ions in the SBF was influenced by its pH and by crevice corrosion. The results of this study elucidate the conditions that lead to the elution of Ti ions in humans, which results in implant corrosion and Ti allergy.

## Introduction

Ti, which is highly biocompatibility and shows excellent resistance to corrosion, owing to the presence of a stable oxide layer, is widely used as an implant material, particularly in patients allergic to other metals [1], [2]. As Ti surfaces oxidize immediately on exposure to air, the degree of Ti ion elution is very small. However, it has been recognized that environmental factors contribute significantly to the increase in the frequency of occurrence of allergic disorders observed worldwide. It is well known that biomaterials release substances that alter the tissue environment to varying degrees. There have been prior reports suggesting that the *in vivo* corrosion of Ti implants gives rise to Ti allergy [3]–[6]. Ti and the other elements released from Ti-based implants have been observed in the tissues and organs near the implants [7]–[10]. However, in spite of several studies involving *in vitro* experiments, the actual biological factors influencing the elution of Ti ions in the human body remain to be clarified [11]–[15].

The purpose of this study was to investigate the oral factors influencing the elution of Ti ions. The conditions within the oral cavity were simulated by immersing Ti specimens in a simulated body fluid (SBF) and in a culture medium in which *Candida albicans* had been grown.

## Materials and Methods

### Preparation of Specimens and Immersion Solution

Commercially pure (CP) Ti (99.5%, Nilaco, Tokyo, Japan), a magnetic alloy (AUM20; containing 18.75–19.50% Cr, 1.75–2.25% Mo, and 0.10–0.30% Ti; Aichi steel, Aichi, Japan), pure nickel (99%≧, Nilaco, Tokyo, Japan), and a gold alloy (Casting gold Type 4; 70% Au, 2% Pt, 3% Pd, 8% Ag, and 16% Cu; GC, Tokyo, Japan) were used. Each specimen was in the form of a plate with the dimensions of 10×10×1 mm. After being polished using waterproof abrasive paper (#800, Sankyo Rikagaku, Saitama, Japan), the specimens were subjected to ultrasonic cleaning in acetone and distilled water for 10 min and then dried.

A simulated body fluid (SBF) was used as the immersion fluid, and its composition was the following: 142 mM Na^+^, 5.0 mM K^+^, 1.5 mM Mg^2+^, 2.5 mM Ca^2+^, 147.8 mM Cl^-^, 4.2 mM HCO_3_^−^, 1.0 mM HPO_4_^2−^, and 0.5 mM SO_4_^2−^ [16], [17]. The pH of the SBF was varied between 2.0, 3.0, 5.0, 7.4, and 9.0 using either hydrochloric or lactic acid.

### Immersion Conditions

#### Immersion of Ti only

Specimens of CP Ti were immersed completely in 50 ml centrifuge tubes (Labcon, San Francisco, California,USA), each a containing 5 ml sample of the SBF having a different pH value (2.0–9.0). The centrifuge tubes were shaken at 80 rpm in a constant-temperature water bath (Personal-11,TAITEC, Saitama, Japan) at 37.5°C, and Ti ion concentrations of the SBF samples were measured 24, 48, and 72 h later. The concentrations were also measured 1, 2, 3, and 4 wk later. To examine the effect on the specimen of the difference in the composition of the SBF solutions, the specimens were immersed in solutions whose pH was adjusted to 2.0 using either hydrochloric or lactic acid.

#### Mechanical stimulus during immersion

Specimens of CP Ti and either 10 alumina balls (with a diameter of 3 mm, Nikkato, Osaka, Japan) or 10 nylon balls (with a diameter of 3 mm, Mochiki, Tokyo, Japan) were immersed completely in a 50 ml centrifuge tubes, each containing a 5 ml sample of the SBF of a different pH value (2.0–9.0) for 72 h. In order to ensure that the wear debris did not affect the Ti ion concentrations of the SBF immersion solutions, the Ti ion concentrations were measured after the immersion solutions had been filtered using a 5 µm pore membrane.

#### Contact between Ti and a dissimilar metal or metallic alloy

To examine the effect of contact between Ti and a dissimilar metal or metallic alloy, specimens of CP Ti and those of the magnetic alloy, gold alloy, and pure nickel were put in contact with each other, with the contact area being either 50 mm^2^ or 100 mm^2^. The in-contact specimens were then immersed in SBF solutions of different pH values (2.0–9.0).

#### Immersion in a culture medium in which *Candida albicans* had been grown

A suspension of *C. albicans* (CAD1), a clinical isolate, was diluted to a concentration of either of 1.0×10^6^ or 1.0×10^5^ colony-forming units (CFU)/ml with the yeast nitrogen base (YNB)/100 mM glucose medium supplemented with 2.5 mM *N*-acetylglucosamine (YNB culture medium). The specimens of CP Ti was immersed in wells of a 24 well plate, with each well containing of 1 ml culture medium, and shaken at 75 rpm and 37.5°C. After 72 h, the Ti ion concentration in the culture medium was measured.

### Measurement and Analysis of the Amounts of Ti ions Eluted

The concentrations of the eluted Ti ions in the different SBF solutions were measured using a polarized Zeeman atomic absorption spectrophotometer (Z-5710, Hitachi, Tokyo, Japan). When the measured value was below the detection limit (5 µg/l), the amount of ions eluted was defined as 0.

The amounts of the eluted Ti ions in the various solutions were statistically examined using ANOVA. This was followed by analyses using Tukey’s test and Mann-Whitney U test (p = 0.05). All statistical analyses were performed using SPSS® (IBM, New York, USA).

## Results

### Immersion of Ti only

**Figure 1:**
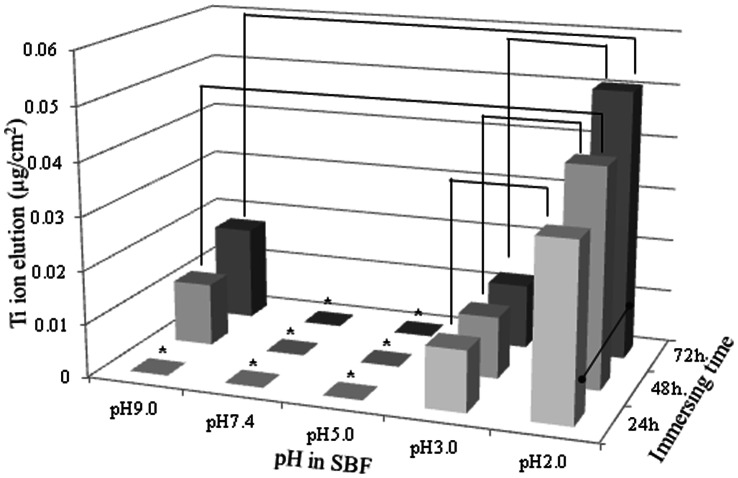
Effect of pH on Ti ion elution from specimens of CP Ti immersed in SBF solutions for 24 to 72 h (*: below detection).

**Figure 2:**
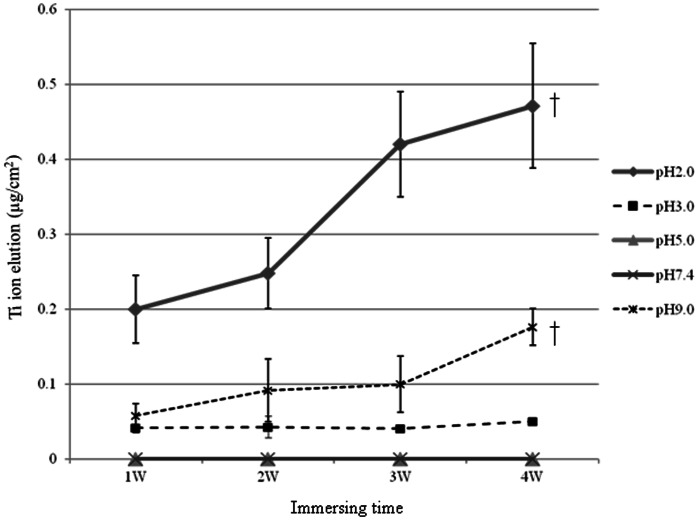
Effect of pH on Ti ion elution from specimens of CP Ti immersed in SBF solutions for 1 to 4 wk. ^†^significant difference among the different immersion times.

**Figure 3:**
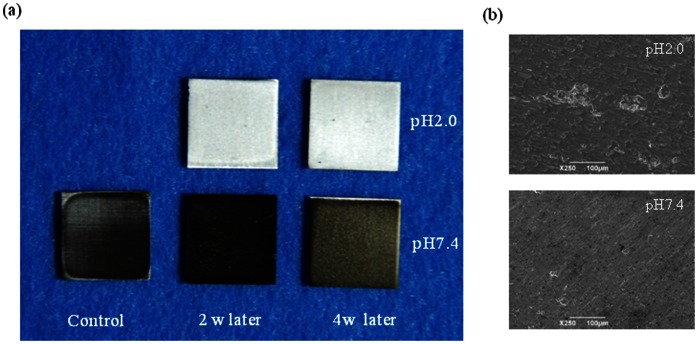
Stereoscopic images of the surfaces of specimens of CP Ti immersed for 2 and 4 wk in SBF solutions(a), SEM image of CP Ti surfaces in immersing for 4 **wk in SBF solutions(b).**

The higher the pH and longer the immersion time were, the greater was the amount of Ti ions eluted. After 24 h, 7.88×10^−8 ^g and 2.74 ×10^−8 ^g of Ti ions were eluted in the SBF samples with pH values of 2.0 and 3.0, respectively. In addition, after 48 h, 2.82×10^−8 ^g of Ti ions was eluted in the SBF solution with a pH of 9.0 (Fig. 1). The amounts of the Ti ions eluted in the SBF solutions with pH values of 5.0 and 7.4 were below the detection limit even after 4 wk. The amounts of Ti ions eluted in the SBF solutions with pH values of 2.0 and 3.0 increased with time for 4 wk. In addition, there were statistically significant differences between the eluted amounts among the groups (Fig. 2). Fig. 3 shows the stereoscopic images of the surfaces of the CP Ti specimens immersed in the SBFs solutions with pH values of 2.0 and 7.4 for two and four wk, respectively. No change was found in the case of the SBF solution with a pH of 7.4. Finally, it was noticed the surface of the specimen of CP Ti immersed in the SBF solution with a pH of 2.0 became lusterless over time.

**Figure 4:**
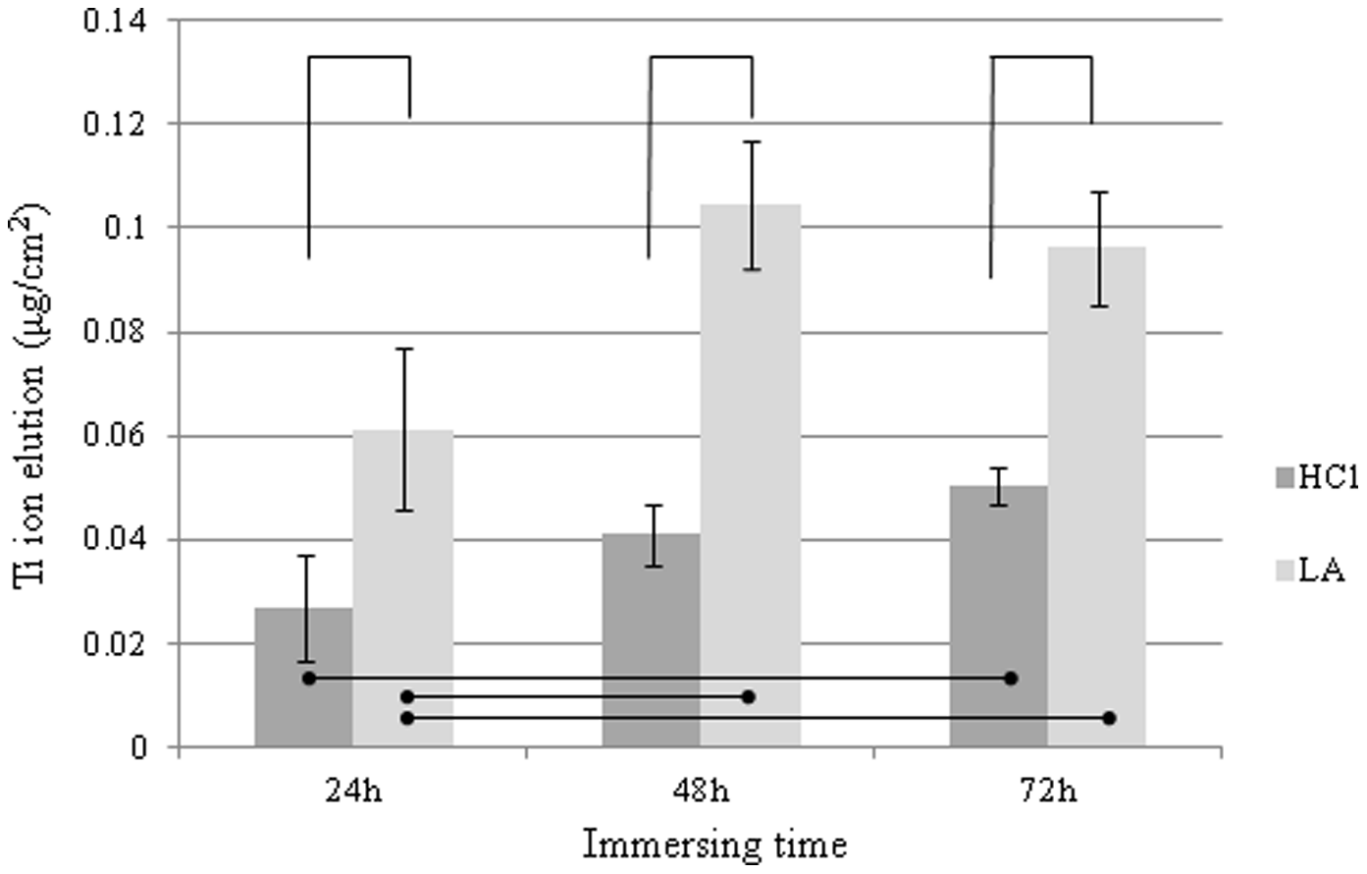
Effect of acid type on Ti ion elution from specimens of CP Ti immersed in SBF solutions (pH = 2) for 24 to 72 h.

The elution of Ti ions in the SBF solutions whose pH values were controlled using lactic acid was significantly higher (by approximately 120% on average) than that in the SBF solutions whose pH values were changed using hydrochloric acid (Fig. 4).

### Mechanical Stimulus during Immersion

**Figure 5:**
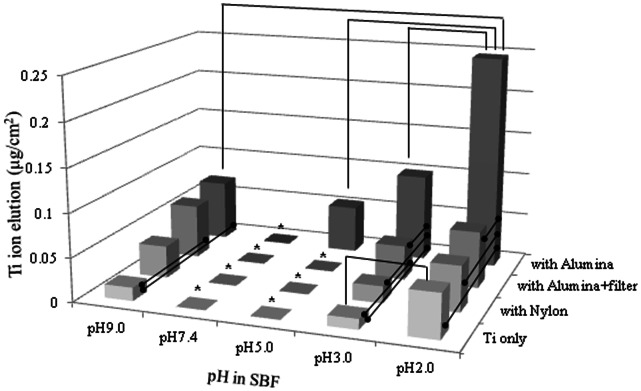
Effect of mechanical stimulus on Ti ion elution from specimens of CP Ti immersed in SBF solutions for 72 h.

Fig. 5 shows the elution of Ti ions from the specimens of CP Ti specimens immersed and shaken for 72 h with either alumina or nylon balls. The elution of Ti ions from the specimens shaken with alumina and nylon balls was 386% and 4% more than that from the Ti specimens that were only immersed in SBF solution with a pH of 2.0 but not shaken with either alumina or nylon balls. When the immersion SBF solutions with a pH of 2.0 were filtered to prevent the wear debris from affecting the measurements, the elution of Ti ions from the specimens shaken with alumina balls was 23% more than that from the specimens shaken with nylon balls.

### Contact between Ti and a Dissimilar Metal or Metallic Alloy

**Figure 6:**
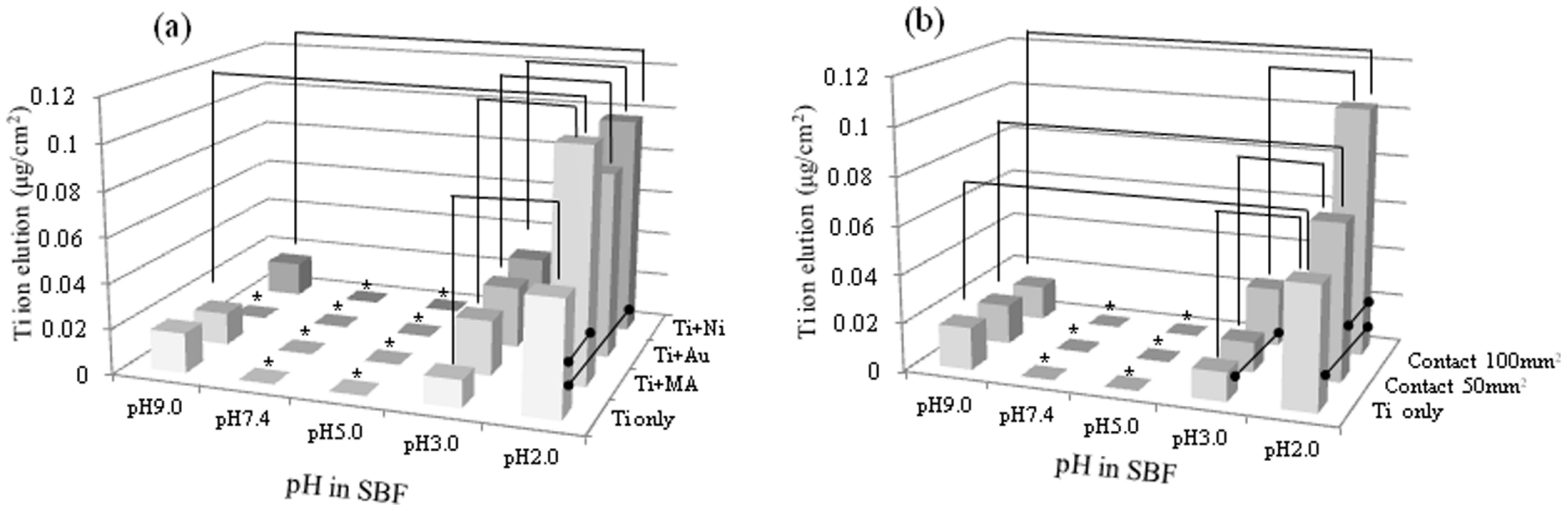
Ti ion elution from a specimen of CP Ti immersed for 72 in an SBF solution and that from a specimen of CP Ti immersed while in contact with a specimen of a dissimilar metal or metallic alloy. (a) shows the effect of the metal or metallic alloy type and (b) the effect of the contact area.

Fig. 6a shows the elution of Ti ions from specimens that were immersed for 72 h while being in contact with a dissimilar metal or a metallic alloy. Being in contact with a dissimilar metal did not have a significant effect on the elution of Ti ions from specimens of CP Ti except with a pH value of 2.0. Fig. 6b shows the elution of Ti ions from specimens immersed for 72 h that were in contact with a magnetic alloy, with the area of contact between the Ti specimens and the magnetic alloy being either 50 mm^2^ or 100 mm^2^. The amounts of Ti ions eluted from the specimens with a pH value of 2.0 when the contact areas were 50 mm^2^ and 100 mm^2^ were 29% and 103% more than that in the case of the not-in-contact Ti specimen, respectively. Significant differences were found between the eluted amounts.

#### Immersion in a culture medium in which *Candida albicans* were grown

**Figure 7:**
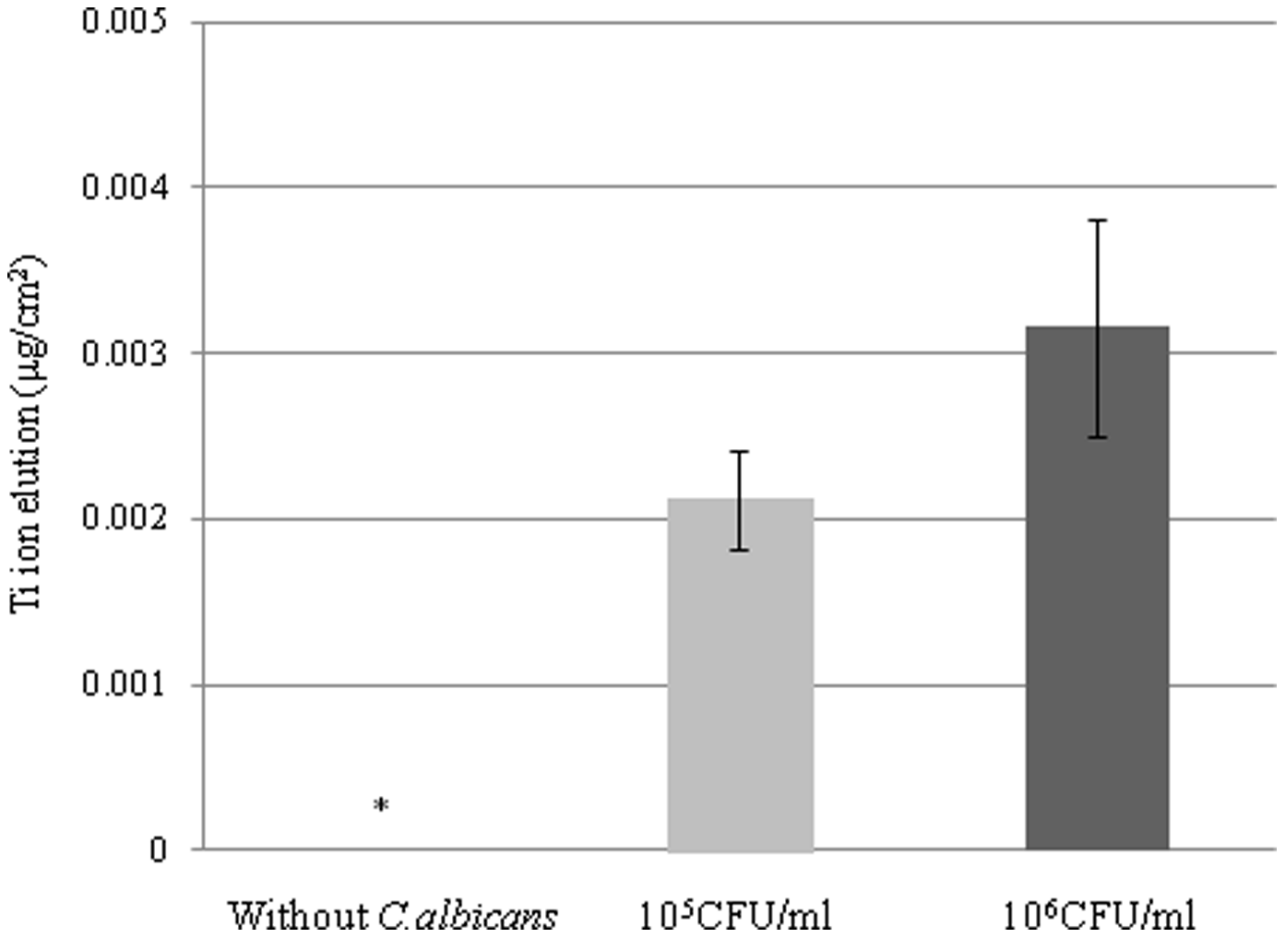
Ti ion elution from specimens of CP Ti immersed for 72 h in a culture in which *Candida albicans* were grown.

Fig. 7 shows the elution of Ti ions from specimens of CP Ti immersed for 72 h in the culture medium in which *C. albicans* had been grown. The elution of Ti ions from the immersed specimens depended on the concentration of *Candida albicans*, but no significant difference was found between the solution with a concentration of 1.0×10^5^ CFU/ml and that with a concentration of 1.0×10^6^ CFU/ml (p = 0.127, Mann-Whitney U-test). The amount of eluted Ti ions when the concentration was 1.0×10^6^ CFU/ml corresponded approximately to that eluted in a SBF solution with a pH of 5.0.

## Discussion

Titanium surfaces can seem stable owing to the formation of an oxide layer on them. Nevertheless, the elution of Ti ions in fluid and the reprecipitation on these ions on the titanium surface occurs repeatedly. Since the corrosion and elution of metals ultimately means an electrode reaction, it is expected that the elution process will be influenced by the pH of the fluid. It is also expected that there will exist a pH value at which the rate of elution increases significantly. However, the mechanism and dynamics behind the elution of the Ti ions in the human body remains unclear. In the present study, the factors that might increase elution in the oral cavity, such as the pH of the surrounding fluid, implant wear, contact with a dissimilar metal, and the presence of microbes, were examined via *in vitro* experiments using an SBF solution.

The results of the study suggest the elution of Ti ions was influenced by the duration of immersion, and the pH of the immersion solution, especially less than pH 3. The oxide layer on the titanium surface will be recovered within several milliseconds even if the oxide layer is accidentally lost, whereas the low pH increases the corrosion owing to anode reaction of titanium.

Body fluid is a buffering solution, and its pH usually remains at 5.2 or higher, even in inflamed regions. However, it is possible for the pH of the fluids in the oral cavity to be temporarily lowered to around 2.0 while drinking and eating [18]. Mu et al. reported that the elution of Ti in a medium contained a culture of macrophages was higher than that in the same medium when it did not contain any macrophages [19]. In addition, it has been reported that the corrosion of implants can also be caused by layers of microorganisms (biofilms). This type of corrosion has been labeled “Biocorrosion” or “Microbiologically Influenced Corrosion (MIC)” [20], [21]. During this kind of corrosion, the local pH becomes extremely low in the areas where the biofilm and the metal are in contact [22]. Thus, even though the conditions involving pH values as low as 2.0 and 3.0, which were investigated in this study, might occur rarely, they do have clinical significance. In order to simulate the above-mentioned condition, specimens of CP Ti were also immersed in a culture medium in which *C. albicans* were grown. After incubation for 72 h, *C. albicans*, which produce acid in large amounts, lowered the pH to 3.0 when present in a concentration of 1×10^5 ^CFU/ml and to 2.81 when present in a concentration of 1×10^6 ^CFU/ml. As a result, elution of Ti ions also increased in the culture medium. This increase was caused by the low pH, the suppression of the formation of the oxide layer by the candidal consumption of oxygen, and crevice corrosion between the candidal biofilm and the titanium surface.

When two metal plates are attached, the interspace between the metal plates becomes susceptible to crevice formation and galvanic corrosion [23]. The elution of Ti from a specimen in contact with a dissimilar metal was significantly higher than that from a not-in-contact Ti specimen. However, there was no difference in the eluted amounts when different metals or metallic alloys were used. This indicated that galvanic corrosion was not the main cause of increase in the Ti ion elution, as has been reported by Reclaru [11]. The larger the contact area between the specimen of CP Ti and that of the dissimilar metal, the higher was the eluted amount. This finding suggests that the increase in the degree of elution was mainly due to crevice corrosion, and not owing to the galvanic effect, as the surfaces of titanium and magnetic alloys are covered by layers of stable oxides.

The elution of Ti ions in the SBF solutions whose pH values were changed using lactic acid was higher than that in the solutions whose pH values were changed using hydrochloric acid. Although it is known that that Ti is highly resistant to corrosion from lactic acid, which contains hydroxy and carboxy groups, can act as a reducing agent. Thus, it is assumed that the lactic acid binds covalently to Ti ions and suppresses the reprecipitation of the ions. As a result, it reduced the stable oxide layer on the specimens of CP Ti, resulting in an increase in the elution of the Ti ions.

It has been reported that the wear factor, which is a measure of the wear of Ti dental implants resulting from the occlusal forces between the implants and the surrounding hard tissue, has an effect on the rate of corrosion of the implant and that higher the wear factor, higher the rate of corrosion [24]. The results of the experiments involving the shaking of the specimens of CP Ti with alumina or nylon balls showed that the elution of Ti ions in the SBF solutions with pH values of 2.0 and 3.0 were higher than those of unshaken (nonstimulated) Ti specimens. However, the net amount of Ti ions eluted decreased when the SBF immersion solutions were filtered before the measurements. There was little significant difference in the amounts eluted from the shaken and unshaken specimens of CP Ti. This was true for all the SBF solutions irrespective of their pH values. It is assumed that the oxide layer on a titanium surface is regenerated under near-neutral conditions if the layer has been removed by mechanical stimulation. Physically stimulating increased the amount of Ti powder produced owing to abrasion, and this, in turn, increased the superficial Ti area, resulting in an slight increase in the amount of Ti ions eluted.

The elution of Ti ions was mainly dependent on the duration of immersion, the pH of the immersion solution, and crevice corrosion. The amount of Ti eluted was one- thousandth that of Ni. The elution of Ti did not take place even after 4 weeks in SBF solutions with pH values of 5.0 and 7.4. However, the amounts of eluted Ti were 7.88×10^−8 ^g in the case of the SBF solution with a pH of 2 and 2.74×10^−8 ^g in the case of the SBF solution with a pH of 3. Watanabe et al injected 7.33×10^−6 ^g (1.25×10^−7^ mol) of Ni initially, and 5.87×10^−8^ g (1.0×10^−9^ mol ) of Ni after 2 wk, into mice to induce a hypersensitivity reaction to Ni [25]. Even though both these amounts induced an allergic reaction to Ni, it is not possible to extrapolate whether the amounts of Ti eluted might induce similar reactions, since the *in vitro* elution of Ti cannot be compared with the *in vivo* elution of Ni.

### Conclusions

The elution of Ti ions in the various SBF solutions was influenced by the pH values of the solutions, particularly for solutions with a pH of <3, and by crevice corrosion. The results of this study would elucidate the conditions that lead to the elution of Ti ions from Ti implants in humans and result in implant corrosion and allergy to Ti.
